# Analgesic Efficacy of Etoricoxib following Third Molar Surgery: A Meta-analysis

**DOI:** 10.1155/2021/9536054

**Published:** 2021-09-09

**Authors:** Lorenzo Franco-de la Torre, Diana Laura Franco-González, Lorena Michele Brennan-Bourdon, Nelly Molina-Frechero, Ángel Josabad Alonso-Castro, Mario Alberto Isiordia-Espinoza

**Affiliations:** ^1^Instituto de Investigación en Ciencias Médicas, Cuerpo Académico Terapéutica y Biología Molecular (UDG-CA-973), Departamento de Clínicas, División de Ciencias Biomédicas, Centro Universitario de los Altos, Universidad de Guadalajara, Tepatitlán de Morelos, Jalisco, Mexico; ^2^Comisión para la Protección Contra Riesgos Sanitarios del Estado de Jalisco, Guadalajara, Jalisco, Mexico; ^3^Departamento de Salud, Laboratorio de Cariología y Medicina Oral, Universidad Autónoma Metropolitana–Xochimilco, Ciudad de México, Mexico; ^4^Departamento de Farmacia, División de Ciencias Naturales y Exactas, Universidad de Guanajuato, Guanajuato, Mexico

## Abstract

**Background:**

The purpose of this meta-analysis was to assess the clinical efficacy of etoricoxib in comparison with traditional NSAIDs for postoperative pain after third molar surgery.

**Methods:**

The quality of studies found in PubMed and Google Scholar was evaluated with Cochrane Collaboration's risk of bias tool. Data on total consumption of rescue analgesics, number of patients using rescue analgesics, global assessment of study treatments, and adverse effects were extracted exclusively from high-quality clinical trials. Each meta-analysis was performed with the Review Manager Software 5.3 for Windows.

**Results:**

The qualitative analysis showed that etoricoxib has better analgesic activity when compared with ibuprofen (2 clinical trials) and diclofenac (1 clinical trial). A similar analgesic efficacy between etoricoxib and nonselective Cox-2 NSAIDs was informed in 3/8 studies (2 compared to ibuprofen and 1 to naproxen sodium). Moreover, the number of patients requiring rescue analgesics in the postoperative period showed a statistical difference in favor of etoricoxib when compared to NSAIDs.

**Conclusion:**

Etoricoxib significantly reduces the number of patients needing rescue analgesics compared to NSAIDs after third molar surgery.

## 1. Introduction

Surgical removal of a mandibular third molar is an important clinical tool to evaluate the analgesic efficacy of new drugs [1, 2]. Surgical injuries on the soft tissue, and particularly trauma on the mandibular bone, produce moderate to severe pain which starts after the anesthetic activity and lasts for several days [1–6].

The most common available drugs to treat dental pain after third molar removal are nonsteroidal anti-inflammatory analgesic drugs (NSAIDs) [7, 8]. The selective enzyme cyclooxygenase-2 (COX-2) inhibitor NSAIDs have similar clinical efficacy as nonselective (COX-2) NSAIDs for the management of osteoarthritis [9] and postsurgical dental pain [10]. Moreover, this type of drug has been related to severe adverse effects, such as myocardial infarction [11–13], acute kidney injury [14], hepatotoxicity [15], and hypersensitivity [16].

Etoricoxib, a relatively new selective (COX-2) NSAIDs, has been used in several clinical studies to control postoperative complications following a third mandibular molar extraction [17–24], and it has shown similar clinical efficacy than nonselective NSAIDs [20, 21, 24].

Recently, a meta-analysis by González-Barnadas et al. [10] showed the clinical efficacy and safety of COX-2 inhibitors versus ibuprofen for relief of postoperative pain after third molar surgery. However, the clinically important analgesic effect of etoricoxib alone following third molar surgery was not evaluated. In addition, that meta-analysis included a small number of clinical trials to assess the effectiveness and tolerability of etoricoxib compared with ibuprofen in oral surgery [10]. Therefore, the purpose of this systematic review and meta-analysis was to evaluate the analgesic effectiveness of etoricoxib versus other NSAIDs in dental science reports.

## 2. Materials and Methods

### 2.1. Literature Search in PubMed and Google Scholar

Both PubMed and Google Scholar were utilized to search for clinical studies using the following keywords: “etoricoxib,” “ibuprofen,” “naproxen,” “diclofenac,” “ketorolac,” “nonsteroidal anti-inflammatory drugs,” “oral surgery,” “dental surgery,” and “third molar surgery.” Article types and language filters (“English” and “Spanish”) were used in PubMed. All clinical trials comparing the clinical effectiveness of etoricoxib and nonselective NSAIDs published up to July 2020 were eligible. This activity was performed by two independent researchers.

### 2.2. Population, Interventions, Control, and Outcome (PICO) Strategy

*Population*: patients undergoing third molar removal.

*Interventions*: etoricoxib administration.

*Control*: cyclooxygenase 2 nonselective NSAIDs.

*Outcomes*: total rescue analgesic consumption, number of patients using rescue analgesics, pain intensity using the Visual Analog Scale (VAS), and global assessment of treatment [25].

The articles that met the specifications of the PICO strategy were turned over for evaluation with Cochrane Collaboration's risk of bias tool.

### 2.3. Risk of Bias Assessment

Quality assessment of each clinical assay was performed with Cochrane Collaboration's risk of bias tool [25–29]. Two independent researchers conducted the full evaluation of each report, and their differences were discussed to obtain a consensus [27–29]. The studies without a high risk of bias were deliberated as high quality (low risk of bias).

### 2.4. Data Extraction

The extracted data were as follows: author, design study, treatment groups, size sample (*n*), dose, total rescue analgesic consumption, number of patients using rescue analgesics, and global evaluation of treatment.

When an article presented two groups of etoricoxib (90 and 120 mg), the events and sample size of the control group were included in the statistical analysis by half to not unrealistically increase the sample size of the combined analysis (i.e., the cases of Brown et al. [19] and Daniels et al. [21]). To do this, the same study reference was used with an added key that allowed the inclusion of the review article by the aforementioned authors on two occasions in the same meta-analysis (Brown et al. [19] and Brown et al. [19–2]; and Daniels et al. [21] and Daniels et al. [21–2]).

### 2.5. Statistical Analysis

The inverse variance statistical method with the standardized mean difference was used to assess the numerical data. Mantel-Haenszel test and odds ratio (OR) were utilized to analyze the dichotomous data. The pooled analysis and forest plot were executed with the Review Manager Software 5.3 for Windows. A *p* value test ≤ 0.05, mean difference, or OR (>1 and within 95% confidence intervals (CIs)) were considered statistically significant [26, 30–32].

## 3. Results

### 3.1. Digital Search

**Figure 1:**
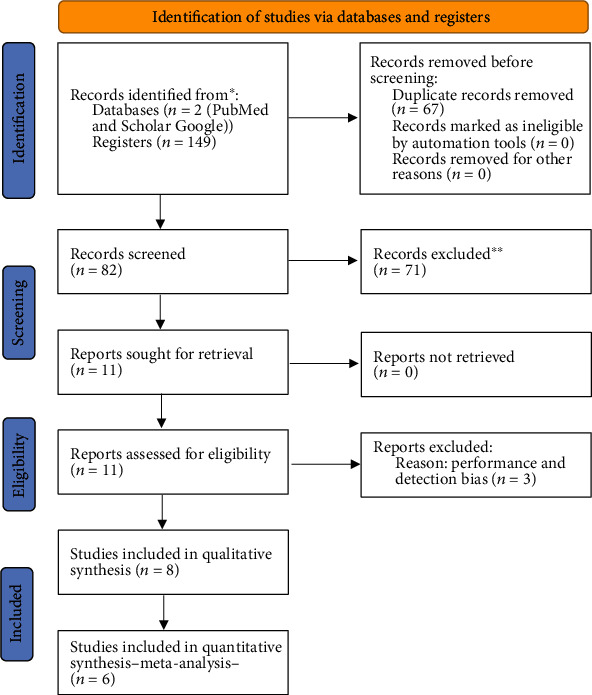
Study flow diagram.

Through both databases, 149 scientific articles were identified. This revision did not include clinical trials using etoricoxib in endodontics or periodontics, as well as those studies comparing etoricoxib with an active control other than NSAIDs [33–37]. After excluding duplicate reports and considering the focus of this review, 11 papers fulfilled the PICO strategy (Figure 1).

### 3.2. Risk of Bias Assessment

**Figure 2:**
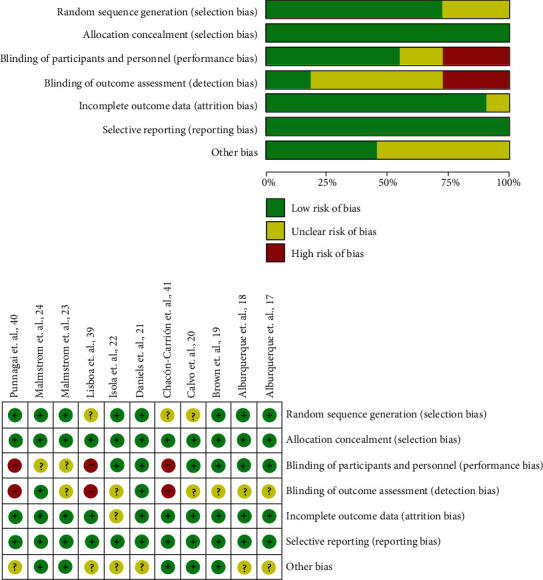
Risk of bias assessment of full text articles.

A total of 8 reports met the quality criteria according to Cochrane Collaboration's risk of bias tool and were used in the qualitative analysis [17–24]. In the quantitative analysis, only 6 articles were included [19–24]. According to Cochrane Collaboration's risk of bias tool, the double-blinded nature was the main problem of the excluded articles [38–40] (Figure 2).

### 3.3. Qualitative Analysis

In line with the quality studies, etoricoxib was compared with ibuprofen in 6 papers, 1 study with diclofenac, and 1 clinical assay versus naproxen sodium. The etoricoxib dose of 120 mg was used in all quality studies in this review (dose range of etoricoxib: 60 to 240 mg). Most studies used a single-dose etoricoxib and a postoperative analgesia approach (Table S1).

According to the conclusions by the authors of each study, the qualitative analysis showed that etoricoxib has better analgesic activity when compared with ibuprofen (2 clinical trials) and diclofenac (1 clinical trial) [19, 22, 24]. A similar analgesic efficacy between etoricoxib and nonselective Cox-2 NSAIDs was informed in 3/8 studies (2 compared to ibuprofen and 1 to naproxen sodium) [20, 21, 23] (Table S1).

### 3.4. Quantitative Analysis: Analgesic Efficacy

**Figure 3:**
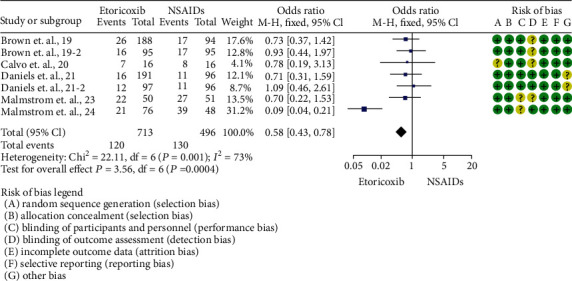
Overall evaluation of the number of patients requiring rescue analgesia.

**Figure 4:**
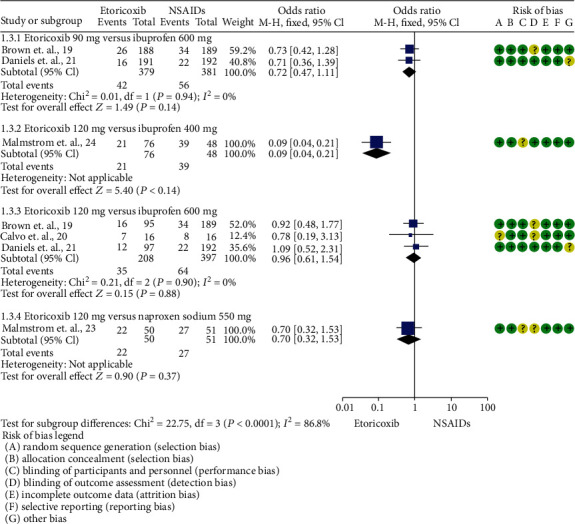
Pooled evaluation according to the etoricoxib dose and the number of patients needing rescue analgesic medication.

Total rescue analgesic consumption was informed only by Calvo et al. [20] (mean difference = −0.44; 95%ICs = −1.38 to 0.5; *p* = 0.36). The number of patients who needed rescue analgesic medication was reported in 5 trials [19–21, 23, 24]. A reduction in the number of patients requiring rescue analgesics was observed in patients who took etoricoxib when compared to NSAIDs (*p* = 0.0004; Figure 3). In this sense, the number of patients needing rescue analgesic medication was lower for etoricoxib in comparison with ibuprofen 400 mg [24] (*p* = 0.00001; Figure 4).

**Figure 5:**
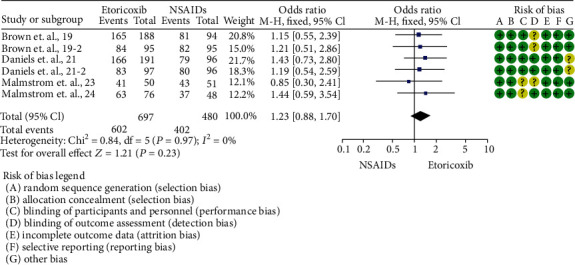
Global assessment of the study medication.

**Figure 6:**
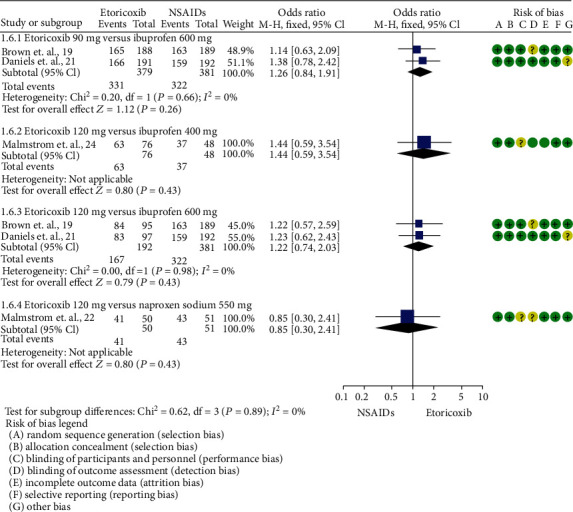
Meta-analysis according to the etoricoxib dose and patient satisfaction.

The global evaluation of the study treatments showed a trend in favor of etoricoxib without a statistical difference (Figures 5 and 6).

### 3.5. Adverse Effects

**Figure 7:**
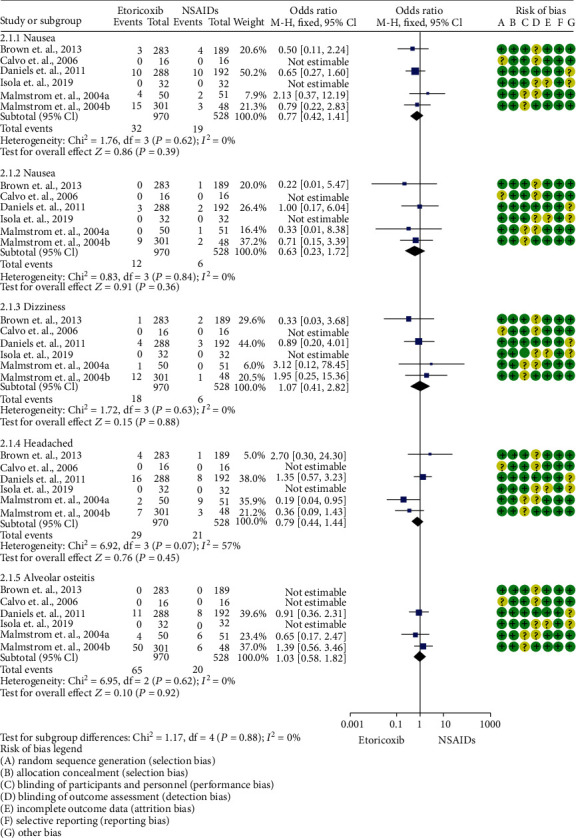
Forest plot of adverse effects associated with etoricoxib and NSAIDs.

The overall adverse effect evaluation of etoricoxib and nonselective (COX-2) NSAIDs was performed using 6 clinical trials [19–24]. The analysis showed no statistical difference (Figure 7).

## 4. Discussion

This is the first meta-analysis to evaluate the individual analgesic effectiveness of etoricoxib in comparison with nonselective (COX-2) NSAIDs following third molar surgery. The most important finding of this review was the lower number of patients who required rescue analgesia in the etoricoxib group when compared with the NSAID group. It should be noted that most indicators of analgesic efficacy were measured dichotomously. For this reason, we could assume that this efficacy evaluation is appropriate [41].

Recently, a meta-analysis by González-Barnadas et al. [10] carried out the pooled analysis of total pain relief (TOPAR), rescue analgesic consumption, and adverse reactions of COX-2 inhibitors versus ibuprofen after third molar removal [10]. In that report, the qualitative analysis included only 3 articles [17, 21, 24] and the meta-analysis just 2 articles [21, 24] because Albuquerque et al. [17] did not provide data for quantitative analysis [10]. The authors concluded that coxibs (also known as COX-2 inhibitors) have an analgesic effect similar to ibuprofen when used in third molar surgery [10]. In other words, the effect of selective COX-2 inhibitors was evaluated globally, and thus, the efficacy of individual coxibs was not known. In our meta-analysis, the assessment of the analgesic effectiveness showed a smaller number of patients requiring rescue analgesics in favor of etoricoxib when compared to NSAIDs after third molar surgery.

The clinical efficacy of etoricoxib in relieving postoperative pain could be explained by the potency with which this agent inhibits the COX-2 enzyme. In vitro tests with whole human blood have described the COX-2 selectivity ratio—(IC_50_ = COX‐1/COX‐2)—of etoricoxib and other NSAIDs as follows: etoricoxib = 106, valdecoxib = 30, celecoxib = 7.6, nimesulide = 7.3, ibuprofen = 0.2, diclofenac = 3, meloxicam = 2, piroxicam = 0.08, and indomethacin = 0.4 [42]. Furthermore, animal studies confirm a superior analgesic potency of etoricoxib compared to other coxibs or NSAIDs. In this sense, we could consider the effective dose 50 (ED_50_) as a measure of drug's potency [43]. Thus, the ED_50_ of etoricoxib was 3.27 mg/kg [44], parecoxib = 1.6 mg/kg [45], celecoxib = 11.58 mg/kg [44], meloxicam = 6.5 mg/kg [45], nimesulide = 7.6 mg/kg [45], piroxicam = 8.5 mg/kg [45], ibuprofen = 58.13 ± 5.32 mg/kg [44], diclofenac = 8.1 mg/kg [45], metamizol = 28.5 mg/kg [45], naproxen = 46.4 mg/kg [45], ketoprofen = 30.3 mg/kg [45], and paracetamol = 225.36 ± 1.02 mg/kg [44] when administered intraperitoneally in the acetic acid-induced abdominal contortions in mice [44, 45].

The assessment of adverse effects by González-Barnadas et al. [10] showed that ibuprofen produced an increased risk of nausea and vomiting compared to COX-2 selective drugs, recommending the use of these latter drugs in patients with a clinical history of gastrointestinal upset. Other systematic reviews and meta-analyses have compared etoricoxib with placebo [46–49]. Aldington et al. [46] found limited clinical evidence of increased cardiovascular risk in patients who took etoricoxib versus placebo. Moreover, the pooled evaluations of adverse reactions from Clarke et al. [47–49] showed a similar risk between etoricoxib and placebo. Baraf et al. [50] assessed the risk of adverse effects of etoricoxib and diclofenac, and the findings showed that etoricoxib had better gastrointestinal tolerability when compared to diclofenac in patients with osteoarthritis. In addition, de Vecchis et al. [51] evaluated 17 clinical trials to analyze the cardiovascular risk of etoricoxib, and the authors concluded that there is no evidence indicating that etoricoxib increases the risk of serious cardiovascular adverse effects when compared to placebo. Zhang et al. [52] assessed different renal events (peripheral edema, hypertension, and renal dysfunction) in 15 clinical trials employing etoricoxib. The authors demonstrated that etoricoxib did not produce any renal alterations. In our meta-analysis, evaluation of minor adverse effects (e.g., nausea, vomiting, dizziness, and headache) showed no statistical differences between etoricoxib and ibuprofen.

The adherence to the PRISMA guidelines, the use of high-quality clinical trials to perform the statistical analysis, and a large sample size are some of the main advantages of our report. On the other hand, the main obstacle of this review and meta-analysis was its retrospective design [53–56].

In conclusion, the number of patients requiring rescue analgesics was lower for etoricoxib when compared to NSAIDs after third molar surgery. Furthermore, according to data extracted from clinical trials with low risk of bias, the safety profiles of etoricoxib and NSAIDs were similar.

## Data Availability

All data are available in the articles included in our manuscript. If you have any further questions feel free to contact me.

## Supplementary Materials

Supplementary Materials Table S1: characterization of the high-quality studies.
