# Supplementary material for: Analgesic Efficacy of Etoricoxib following Third Molar Surgery: A Meta-analysis
Source: Behav Neurol. 2021 Sep 9;2021:9536054. doi: 10.1155/2021/9536054 (PMC8445708; doi:10.1155/2021/9536054)
Supplement: Supplementary Materials — Table S1: characterization of the high-quality studies. [file 9536054.f1.pdf]

Table S1. Characterization of the high-quality studies.

First author and study design	Treatments (n) and type of strategy for pain control	Patients, surgical procedure, and rescue analgesic	Evaluation of the clinical efficacy	Loss to follow-up	Conclusion
Albuquerque et al (17). Randomized, triple-blind, crossover clinical study.	Group A: Etoricoxib 120 mg 1 h before surgery (n = 24).<br>Group B: Ibuprofen 400 mg 1 h before surgery (n = 24).<br>Group C. Placebo 1 h before surgery (n = 24).<br>Pre-emptive analgesia.	Healthy patients (ASA I).<br>Third molar surgery.<br>Ibuprofen 300 mg.	The visual analog scale (VAS) was used for the evaluation of pain at 0, 2, 4, 6, 8, 10, 12, 24, 48, and 72-h and 7-days post-extraction.<br>Total consumption of rescue analgesics.<br>Adverse effects were not evaluated.	There were no losses to follow up.	The conclusion did not include the analgesic efficacy of treatments.
Albuquerque et al (18). Randomized, triple-blind, crossover clinical study.	Group A: Etoricoxib 120 mg 1 h before surgery (n = 5).<br>Group B: Ibuprofen 400 mg 1 h before surgery (n = 5).	Healthy patients (ASA I).<br>Third molar surgery.<br>No rescue analgesic medication was used.	VAS was used for the evaluation of pain at 0, 2, 4, 6, 8, 10, 12, 24, 48,	No losses were reported.	The conclusion did not include the analgesic efficacy of treatments.

	Group C. Placebo 1 h before surgery (n = 5).<br>Pre-emptive analgesia.		and 72-h and 7-days post-extraction.<br>Adverse effects were not assessed.		
Brown et al (19).<br>Randomized, double-blind, parallel, clinical trial.	Group A: Etoricoxib 90 mg once daily for three days (n = 188).<br>Group B: Etoricoxib 120 mg once daily for three days (n = 95).<br>Group C: Ibuprofen 600 mg every 6 h for three days (n = 189).<br>Group D: Acetaminophen 600 mg / Codeine 60 mg every 6 h for three days (n = 56).<br>Group E: Placebo for three days (n = 45).<br>Post-operative analgesia.	Healthy patients (ASA I).<br>Third molar surgery.<br>Acetaminophen 325 mg.	Recall of average and worst pain.<br>Patient global assessments using a 4 categorical scale (where 0= poor and 4= excellent).<br>Use of rescue medication.<br>Incidence of adverse effects.<br>All these variable were evaluated a 24 and 48 h after surgery.	Group A = 3.<br>Group B = 2.<br>Group C = 3.<br>Group D = 6.<br>Group E = 1.	Etoricoxib was superior for the management of pain than ibuprofen following third molar removal.

Calvo et al (20). Randomized, double-blind, crossover, study.	Group A: Etoricoxib 120 mg once daily for four days (n = 16). Group B: Ibuprofen 600 mg every six h for four days (n = 16). Post-operative analgesia.	Healthy patients (ASA I). Third molar surgery. Acetaminophen 750 mg.	VAS was used for the evaluation of pain at 0, 24, 48, 72, and 96 h following surgical removal. Total consumption of rescue analgesics. Incidence of adverse effects.	No losses were reported.	Similar efficacy of etoricoxib and ibuprofen for control of pain and trismus after third molar surgery.
Daniels et al (21). Randomized, double-blind, parallel, clinical trial.	Group A: Etoricoxib 90 mg once a day (n = 191). Group B: Etoricoxib 120 mg once a day (n = 97). Group C: Ibuprofen 600 mg every six h for 24 h (n = 192). Group D: Acetaminophen 600 mg / Codeine 60 mg every six h for one day (n = 62).	Healthy patients (ASA I). Third molar surgery. Hydrocodone bitartrate 5 mg / acetaminophen 500 mg.	Total pain relief at 6 h (TOPAR6), overall evaluation at 24 h, onset of analgesia, peak analgesic action, duration of analgesic activity, and incidence of adverse events.	No losses were reported.	Etoricoxib 90 mg and 120 mg produced similar efficacy than ibuprofen for the control of postoperative pain following dental surgery.

	Group E: Placebo every six h for a day (n = 46).<br>Post-operative analgesia.				
Isola et al (22).<br>Randomized, triple-blind, crossover clinical study.	Group A: Etoricoxib 120 mg twice a day for five days (n = 32).<br>Group B: Diclofenac 50 mg twice a day for five days (n = 32).<br>Group C: Placebo twice a day for five days (n = 33).<br>Post-operative analgesia.	Healthy patients (ASA I).<br>Third molar surgery.<br>Rescue medication was not indicated.	VAS was used for the evaluation of pain at 30 minutes, 2, 6, 12, 24, 48 h, 7 and 10-days after dental surgery.<br>The peak postoperative pain score.	Losses were not reported.	Etoricoxib showed better analgesic activity than diclofenac and placebo after third molar removal.
Malmstrom et al (23).<br>Randomized, double-blind, parallel, clinical trial.	Group A: Single oral dose of etoricoxib 120 mg (n = 50).<br>Group B: Single oral dose of naproxen sodium 550 mg (n = 51).<br>Group C: Single oral dose of acetaminophen 600 mg / codeine 60 mg (n = 50).	Healthy patients (ASA I).<br>Third molar surgery.<br>Acetaminophen 500 mg / hydrocodone 5 mg.	TOPAR8, overall assessment scale at 8- and 24-h, patients needed to take recue analgesics in 24 h post-extraction, onset of analgesia, peak analgesic action, duration of analgesic activity, and	Losses were not reported.	Similar analgesia was observed for etoricoxib and naproxen sodium after the extraction of third molars..

	Group D: Single oral dose of placebo (n = 50). Post-operative analgesia.		incidence of adverse reactions.		
Malmstrom et al (24). Randomized, double-blind, parallel, clinical trial.	Group A: Single oral dose of Etoricoxib 60 mg (n = 75). Group B: Single oral dose of etoricoxib 120 mg (n = 76). Group C: Single oral dose of etoricoxib 180 mg (n = 74). Group D: Single oral dose of etoricoxib 240 mg (n = 76). Group E: Single oral dose of ibuprofen 400 mg (n = 48). Group F: Single oral dose of placebo (n = 49). Post-operative analgesia.	Healthy patients (ASA I). Third molar surgery. Acetaminophen 500 mg / hydrocodone 5 mg.	TOPAR8, overall assessment scale at 8- and 24-h, patients needed to take recue analgesics in 24 h post-extraction, onset of analgesia, peak analgesic action, duration of analgesic activity, and incidence of adverse reactions.	There were no losses to follow-up.	Etoricoxib had better pain management than ibuprofen after dental surgery.
